# Attitudes of employers towards people with visual impairment: a scoping review

**DOI:** 10.3389/fresc.2024.1383984

**Published:** 2024-11-19

**Authors:** Claire L. Castle

**Affiliations:** ^1^ Social and Welfare, BRAVO VICTOR, London, United Kingdom; ^2^ School of Music, Faculty of Arts, Humanities and Cultures, University of Leeds, Leeds, United Kingdom

**Keywords:** visual impairment (VI), visual impairment & blindness, employment, equitable access, disability

## Abstract

This scoping review explored literature relating to employer attitudes towards employees and candidates with visual impairment (VI). Systematic searches identified 17 relevant articles published since 2018. Thematic synthesis highlighted findings relating to several themes: explicit and implicit attitudes of employers; employer concerns, including those relating to employee performance, and the experiences of both customers and colleagues; and factors which might impact on employer attitudes, such as gender and prior experience of having hired someone with VI. Findings indicate a tendency for employers to possess negative or, at best, neutral attitudes towards people with VI, and the central role that improved knowledge of VI and the capabilities of people with VI may play in generating positive employer attitudes. The review highlights the need for further exploration of this topic, particularly given the limited geographical spread of recent research, and a lack of consideration of the shared experience of employer and employee.

## Introduction

Despite anti-discriminatory policy and legislation across Europe and beyond (e.g., (1)), which aim to create an inclusive and accessible labour market, global research shows that employment remains a key area of life in which people with disability remain disadvantaged (2). In the UK, the Department for Work & Pensions (3) reported a disability employment gap of 28.9%, with the employment rate for those with no disability reported at 82.5%, compared to 53.6% for people with no disability. Notably, individuals with visual impairment (VI) appear to fare particularly poorly in terms of employment. Figures from the Office for National Statistics (4) suggest that the employment rates for those with self-reported “difficulty seeing” as their main impairment was poorer in 2021/2022 (42.4%) than for those with other types of disability, including difficulty hearing (67.7%) and disability associated with arms and hands (60%). Research highlights the multiplicity of employment challenges experienced by people with VI which, as in the social model of disability (5), often reflect engrained and negative societal attitudes regarding individuals with disability. The model views disability as a social construction based on the relationship between the person with an impairment and a disabling society (5). Whilst contended and updated many times since Shakespeare's (6) seminal writing on the model [see Oliver (7) for a discussion], it remains useful in highlighting the impact of structural and attitudinal barriers on employment experiences, which have led to the continued segregation of workers with disability (8).

Indeed, across both academic and grey literature, it is employer attitudes that have most consistently emerged as one of the biggest perceived barriers to employment for people with VI (9–11). Research undertaken for the RNIB (12), for example, found that the attitudes of employers preventing access to job opportunities was felt to be the biggest employment-related barrier amongst 656 blind and partially sighted survey respondents. People with VI have reported negative and discriminatory attitudes from employers (13), inaccurate assumptions about their needs and abilities (14), and the challenge of overcoming stereotypes and proving competencies as a candidate with VI (12).

Other barriers to employment reported by those with VI have included inaccessible job application processes (15), concerns regarding disclosure of an impairment to employers (16), difficulties associated with getting to work (12), and technological challenges in the workplace (17). Sources of support, such as Access to Work, have been identified as beneficial, but evidence suggests some limitations of the scheme, including slow application processing, the need to wait for financial reimbursement (18), and low uptake amongst those in employment (19).

Despite evidence of continued barriers to employment for people with VI, the attitudes of employers towards these individuals has yet to be fully explored. As McDonnall and Antonelli (20) write, attitudes are important due to their potential influence on behaviours; if employers hold negative attitudes towards people with VI, it can be assumed that people with VI may experience discrimination during hiring and employment processes. The current review addresses the research question “*What does existing literature tell us about the attitudes of employers towards employees or potential employees with visual impairment?*”. Understanding employer attitudes, and how negative attitudes might be improved, may be an important step towards improving employment outcomes for people with VI. As a scoping review, this article seeks to outline existing research, offering a means to identify gaps in the existing literature (21). This approach allows breadth of exploration where a literature base may be relatively small; this was the case with the current topic, which has primarily been explored from the perspective of employees.

## Methods

**Figure 1:**
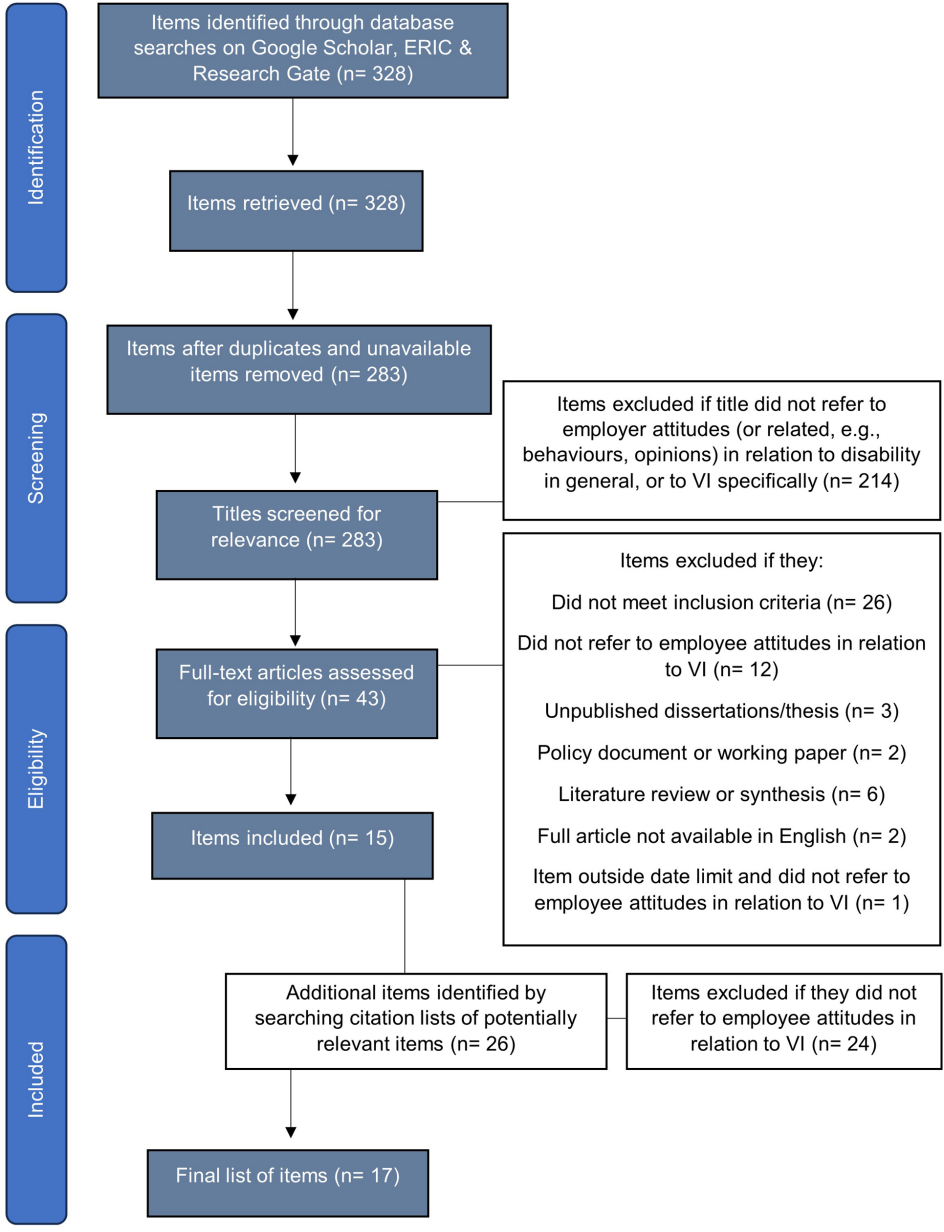
PRISMA flow diagram illustrating the search strategy. This flow diagram provides the phases of article identification and selection, resulting in the identification of 17 items deemed eligible for inclusion in the review.

The methodological framework followed five stages outlined by Arksey and O’Malley, and further discussed and expanded by Levac et al. (22): (1) Identify the research question; (2) Identify relevant studies; (3) Study selection; (4) Charting the data; and (5) Collating, summarizing, and reporting results. Levac et al.'s (22) proposed additional stage of consultation was not included given the focus of the current review on exploring current literature. In line with guidance from Peters et al. (23) on the reporting of scoping reviews, Figure 1 provides a PRISMA Flow diagram outlining the results of the search strategy.

### Identifying the research question

The review process began with clear articulation of the research question, which aimed to explore what literature tells us about the attitudes of employers towards employees or potential employees with VI. This research question was informed by previous research experience of the author on the topic of employment for people VI, reports from individuals with VI of attitudinal barriers to employment, and the limited knowledge surrounding this topic in the existing literature. In line with the wider aims of scoping reviews, this research question was broad enough to examine the extent, range, and nature of the research available, whilst focusing on this specific aspect of the employment experience for people with VI.

### Identification of relevant studies

The second stage was the identification of relevant studies. An initial search of the literature helped to establish key search terms for use in the full search. No limits were set on study design or country of origin but there was a date restriction imposed to only include items published from 2018 onwards; this aimed to provide an up-to-date insight of the topic of greatest relevance to current employment experiences for people with VI. A search of Google Scholar was undertaken on 6th January 2024 using the words: “employer” OR “employers” AND at least one of the following: “attitudes” “perceptions” “visually impaired” “vision impairment” “sight loss” “sight impaired” “visual impairment” “blind” “partially sighted” “partial sight” and “disability”. This search returned a total of 327 items. Additional searches with these terms were carried out through ERIC and ResearchGate on the 10th and 11th January, respectively; these searches were checked for relevant titles which returned just one additional source not previously identified (totalling 328 items).

### Selecting studies

The titles of the 328 items were appraised for their potential relevance to the research question (i.e., whether they referred to employer attitudes or perceptions in relation to VI, or disability more generally). Those articles which were considered potentially relevant were downloaded, or bookmarked, if not available. Those titles for which the content was not clear were retained for checking at the next stage of the process. Items were excluded if they were duplicates, if the full text was not available, if they were not peer-reviewed, not available in English, or if they were reviews and/or only synthesised existing literature or evidence. Sources were included if the data reported related to people with VI, or people with disability more generally, only if specific reference was made to experiences with, or perceptions of, people with VI. Note, the term “employer” refers to anyone working in a position that might determine the employment outcomes for people with VI. In the current review this includes HR/hiring managers, high-level management/executives, and high-level administrators with hiring authority.

The abstracts of the remaining 43 items were read, and items excluded if they were not of relevance to the research question. Fifteen sources were retained, which either focused on attitudes or perceptions of employers towards VI and/or employment of people with VI, or on disability, but reported results relating specifically to VI. The reference lists of these items, and those excluded, but relevant to the topic of employment and disability, were hand-searched for further sources. Twenty-six items were identified as potentially relevant but, following abstract review, just two of these were retained.

### Charting the data and reporting results

The final list of 17 items were read in full, and relevant material from each was sorted according to key topics, ready for the fifth, and final stage of collating, summarising and reporting findings. An overview of the sources and their findings can be found in Table 1 and a narrative discussion of key topics identified is provided below.

**Table 1:** Overview of included sources.

Author, year, and country	Study design	Population	Key findings	Limitations
Papakonstantinou and Papadopoulos, 2020 (24) Greece	Survey (*N* = 196).	196 private sector employers. None had VI, nor new someone with VI in their immediate family,	• 36.6% of employers expressed a positive response towards hiring individuals with VI and 24.7% a negative response. • 74.8% had a negative response regarding intentions to hire someone with VI on a full-time rather than part-time basis. • 63.5% had a negative response regarding intentions to develop job roles for employees with VI. • 28.4% indicated negative responses regarding offering employees with VI the same development opportunities as other employees. • 58.6% demonstrated intentions to participate in a funded programme to create appropriate infrastructure for individuals with VI. • 48% gave positive responses regarding intentions to attend a seminar about integrating individuals with VI; 69.5% expressed positive intentions to undertake actions to support this integration.	Unvalidated scale; sample drawn from only one city; insight limited to quantitative rating scales of attitude.
McDonnall and Crudden, 2018 (25) USA	Survey (*N* = 379).	379 people with hiring authority in a company (e.g., managers, executives and HR),	• Five variables significantly predicted employer attitudes regarding people with VI: having hired someone with VI in the past, knowledge about how work tasks can be accomplished, belief in knowledge, having a relationship with vocational rehabilitation (VR), and being female. • Having hired someone with VI functioned as a mediator of the relationship between communication with VR and employer attitudes, indicating that communication with VR may influence employers’ hiring decisions. • The average employer attitude score was 34.03 (SD = 13.49), with scores ranging from 0 to 66. The average score on the knowledge scale was 0.25 (SD = 0.60), with scores ranging from 0 to 3. 82.3% of respondents did not know how any of the work tasks listed could be performed by an employee with VI.	Self-selection bias (e.g., high percentage of employers who had hired someone with VI, 37.2%).
McDonnall and Cmar, 2022 (26) USA	Survey (*N* = 387).	387 managers or high-level administrators with hiring authority,	• The five predictors of employer attitudes were: awareness of people with disabilities at work, knowledge, inaccurate belief in knowledge, previous hiring of someone who was blind/VI, and having a personal relationship with someone blind/VI. • Previous communication with vocational rehabilitation (VR), having a company policy about hiring people with disabilities, and personal relationship predicted having hired someone who was blind/VI. • Respondents who worked for companies that had a policy about hiring people with disabilities had 4.85 times higher odds of being aware of people with disabilities at their company than those whose did not. • The odds of hiring a person who was blind/VI were 5.61 times higher for those who communicated with VR, compared to those who did not. • The odds of hiring were 3.80 times higher when there was a company policy for hiring people with disability than when there was not, and 3.37 times higher when the employer had a personal relationship with someone who was blind/VI than when they did not.	Potential self-selection bias; not possible to infer causality in associations between attitudes and hiring behaviours.
McDonnall and Antonelli, 2018 (20) USA	Online survey (*N* = 343) including the IAT-BVI and EABES.	343 hiring managers,	• The mean *D* score on the IAT-BVI was 0.76 (SD = 0.40) (zero would indicate no automatic association for Blind/Negative or Sighted/Positive). Employers had strong negative implicit attitudes about the competence of people who are blind (they more easily, or automatically, associated competence with sight and incompetence with blindness). • Implicit attitudes were not associated with personal characteristics, exposure to people who are blind, or explicit attitudes, but were associated with knowledge about how blind people perform work tasks and, for employers who had hired a blind person, performance ratings of those employees (employers who rated their blind employee's performance as above average had significantly more positive implicit attitudes than employers who rated it as average). • The mean score for the EABES was 34.49 (SD = 12.87).	Limitations in matching of photographs; use of the language “blind” may have been interpreted differently by employers.
McDonnall and Antonelli, 2019 (27) USA	Online survey (*N* = 388).	388 hiring managers,	• All of the variables explored except gender had a significant univariate relationship with having hired someone with VI (having a personal relationship with someone who is VI, employer attitudes, knowledge, belief in knowledge, prior communication with vocational rehabilitation, VR, having a relationship with VR, having received an application from someone with VI, company size, and having a company policy on hiring people with disability). • Prior communication with VR was a strong predictor of hiring behaviour; those who had communicated with VR were 4.3 times more likely to hire someone with VI. • Employers from large companies, and employers from companies with policies on hiring people with disability, were more likely to hire someone with VI. • Employer attitudes remained a significant variable in the model even after receipt of an application was included, demonstrating the barrier created by negative employer attitudes. Statistical results found that the small proportion who did not hire an applicant with VI after receiving an application had more negative attitudes. • Two models were tested. In Model 1, variables that were significantly associated with hiring behaviour were prior communication with VR, employer attitudes, company size, company policy, and having a personal relationship with someone with VI. In Model 2, significant variables were receiving an application, employer attitudes, and a personal relationship with someone with VI.	Potential self-selection bias; not possible to infer causality due to cross-sectional nature of study.
McDonnall, 2018 (28) USA	Online survey (*N* = 379).	379 people with hiring authority within a company (e.g., managers, executives and HR),	• Ten independent variables were included in a logistic regression model to predict employer hiring decisions (employer attitudes, gender, high income, college graduate, knowledge of VI, belief in knowledge relating to VI, communication with vocational rehabilitation, VR, relationship with VR, having a personal relationship with someone with VI, and being a large employer). The model was statistically significant but only two of the 10 variables significantly predicted hiring decisions: communication with vocational rehabilitation (VR) and employer attitudes. • Employers who had communicated with VR were 24.1 times more likely to have hired a person with VI compared to those employers who had never communicated with VR. • A 10-point higher score on the EABES resulted in odds 2.61 times higher of having hired someone with VI.	Not possible to infer causality in associations between attitudes and hiring behaviours due to cross-sectional nature of the data (e.g., whether attitudes impacted on hiring, or vice versa).
McDonnall and Antonelli, 2020 (29) USA	Intervention study (*N* = 59) with pre-, post- and 4-month follow-up survey.	59 hiring managers at a large financial services company who had not previously hired a person with VI,	• A meeting between a vocational rehabilitation (VR) professional and a hiring manager improved employers’ attitudes, knowledge, and intent to hire. This was regardless of the approach used (educational or DCA, dual customer approach) or vision status of the VR professional (blind or sighted). • The educational approach resulted in increases in knowledge that were retained at follow-up, while the dual customer approach did not. • Improvements in intent to hire were present post-intervention but not retained at follow-up, suggesting that ongoing contact between educators and employers may be required to positively impact the hiring of people with VI.	Self-selecting sample of employers from one workplace; knowledge measure may not have represented general overall knowledge of VI; only explored the impact of one single meeting on attitudes, thus lacks external validity.
McDonnall and Antonelli, 2022 (30) USA	Intervention study (*N* = 57) with pre- post- and 4-month follow-up survey.	57 hiring managers [as in McDonnall and Antonelli (29)],	• A meeting between a vocational rehabilitation (VR) professional and a hiring manager impacted on hiring manager's implicit attitudes. IAT-BVI scores decreased significantly after the meeting (the size of the change was small). Compared to pre-test, differences were slightly larger at the 4-month follow-up than at post-test, indicating that the average effect of the intervention across conditions did not diminish over time. • Type of approach (educational or DCA, dual customer approach) and vision status of VR professional (blind or sighted) were not significantly associated with IAT-BVI change. However, follow-up analyses found that participants who met with the blind VR professional had a significant decrease in IAT-BVI score and participants who met with a sighted professional did not exhibit a significant decrease in scores.	Self-selecting sample of employers from one workplace; small subgroup sizes impacting on statistical power; only explored the impact of one single meeting on attitudes, thus lacks external validity.
McDonnall et al., 2019 (31) USA	Online survey (*N* = 322) including the IAT-BVI.	322 sighted “blindness professionals” and 450 employers,	• Blindness professionals exhibited a slight association, whereas employers exhibited a strong association, for Sighted with Competence and Blind with Incompetence. • Blindness professionals’ mean score on the IAT-BVI was 0.30 (SD = 0.49). Employers’ mean score on the implicit attitudes test was much higher, 0.76 (SD = 0.39). Blindness professionals and employers had statistically significant differences in implicit attitudes. • Follow-up analysis dichotomized blindness professionals into those that had worked in the field for less than 17 years vs. 17 years or more. The difference between the groups was statistically significant; blindness professionals’ experiences with people who are blind during their careers may play a role in shaping more positive attitudes than those held by others.	Potential self-selection bias; study only considered implicit attitudes of those with experience of working with people with VI; professionals or employers with VI were not included.
McDonnall and Lund, 2020 (32) USA	Online survey (*N* = 388).	388 hiring managers,	• The use of the Theory of Planned Behaviour (TPB) to explain employers’ hiring intentions of people who are blind or VI was tested, finding that the proposed TPB structural model provided good data fit. • Attitudes about productivity, subjective norms, and perceived behavioural control accounted for more than 61% of the variance in intent to hire people who are blind or VI. • Attitudes about productivity of a blind employee had the strongest relationship with intent to hire, followed by subjective norms and perceived behavioural control.	Potential self-selection bias; instructions were added regarding intention to hire (i.e., employers instructed to assume that they had a qualified applicant) which may have inflated intent to hire scores; some participants may have missed this statement, resulting in potentially different answers; study explored intent to hire, not actual hiring behaviours.
Eckhaus, 2022 (33) Israel	Online survey (*N* = 975) with open-ended response questions and one Likert-style closed response question.	975 Employers with hiring authority,	• The hypothesized model for predicting decision-making relating to hiring someone with VI was a good fit. Financial incentives directly and positively affected employers’ attitudes toward hiring someone with VI. Financial incentives were found to mediate information provision and job match with employers’ attitudes. • Qualitative interviews provided examples relating to the potential impact of evidence relating to candidate abilities, financial incentives to hire candidates with VI, and the mediating role of financial incentive in both job match and attitudes and information and attitude. • Results highlight the importance of financial incentives as the “bottom line” from employers’ perspectives regarding candidates with VI (regardless of appropriate job position or relevant information regarding how they will fulfil a job role).	Lack of data on what employer attitudes were; attitude measured by one single quantitative item, lacking nuance; focus of question on an employer or business, rather than the individual themselves and their opinions and experiences.
Eckhaus and Krisi, 2022 (34) Israel	Online survey (*N* = 1,036) to inform the development of a 6-item psychometric measure.	1,036 managers with hiring authority,	• Responses indicated generally negative responses regarding the employment of individuals with VI. The majority of respondents (*n* = 485, 46.8%) rated the statement “Organizations/employers would prefer employing a person without a disability over a visually impaired or blind person” at 5 (where 1 is strongly disagree and 5 is strongly agree). • Exploratory Factor Analysis (EFA) followed by Confirmatory Factor Analysis (CFA) was used to develop a model of employer attitudes towards people with VI, to develop a measurement tool. CFA showed good fit to the observed data and the tool developed may be useful.	Unclear from which workplace managers were recruited and/or their experience of working with people with VI. Frequency/mean are not reported for the items used (except for one)
Østerud, 2023 (35) Norway	Field experiment (*N* = 145) Follow-up interviews (*N* = 18)	145 employers in field experiment. Interviews with 18 employers (14 general managers and four HR managers),	• Qualitative insights showed that cultural “fit” may be a concern for employers regarding employees with VI. Talking about one employee taken on through a wage subsidy scheme, who had a mobility and visual impairment, one manager said: • “He was in the public employment system and because of this he became aware of his challenges in a way that I think was negative. There was a lot of fuss about nothing. […] So, he just didn’t fit into the group, culturally, with people who are used to getting by on their own and not complaining as much, and they kind of got someone the opposite of that. And that was, that is a challenge.” • Findings suggest that some employers do not consider people with a disability to be equally equipped to participate socially due to inaccessible environments, along with a lack of cultural “fit” and/or a perceived lack of social aptitude.	Limited insight into experiences with employees with VI (just one stated example); interviews unsuitable for investigating implicit attitudes and relied on recall of past decision-making processes.
Berre, 2023 (36) Norway	Factorial survey (*N* = 1,341) evaluating fictional job-seeker profiles.	11,939 vignettes were evaluated by 1,341 employers,	• Disabled job seekers were assessed as less employable than non-disabled job seekers. Non-disabled job seekers had a predicted score of 4.9 on the hiring assessment scale (on average, moderately likely to be hired). Job seekers with a disclosed impairment had, on average, a predicted score 2.6 points lower than this. • There were significant differences between the average predicted score of job seekers with different types of disabilities. Blind people were assessed as the least employable, scoring a mean of 1.6 on the hiring assessment scale. Blind people were assessed significantly lower than all other types of disability (wheelchair users, hearing impaired, intellectually disabled, chronically/mentally ill).	Assessments of job-seeker profiles may have limited transferability to real-life decision-making processes (e.g., limited details about candidate); potential effects of social desirability bias, if employers became aware of participating in a study on disability recruitment.
Fyhn et al., 2021 (37) Norway	Survey (*N* = 1,230) with vignettes.	305 supervisors and 925 employees,	• Vignette characters describing mental health issues and physical disabilities (except for auditory impairment) were less likely to be assessed positively than the reference case; cultural minorities were assessed as positive, or more positively than the reference case. • The vignette describing VI was least likely to be rated positively; just 17% rated this vignette positively (70% rated the auditory impairment vignette positively). • Few respondents had previous experience with colleagues with VI (*n* = 852, 8%). • The most cited free-text theme for the character with VI (72%) was “Nature of the work” (e.g., “selling products with visual details will be challenging”). For VI, accommodation was by far the most frequently cited barrier.	Assessments of job-seeker profiles may have limited transferability to real-life decision-making processes (e.g., limited details about candidate); potential effects of social desirability bias, if employers became aware of participating in a study on factors impacting on employee desirability.
Ebrahim et al., 2022 (38) South Africa	Case study (*N* = 2) with qualitative interviews.	2 employers (of employees with disability who had recently attended a computer literacy training course to facilitate employment for people with disability),	• *Theme 1: Equal but Different- Values and Obligations* (e.g., specific guidelines and legislative mandates for appointing people with disabilities; disability and the notion of being disadvantaged are either similar or interchangeable; despite being given preference, people with disabilities are “measured with the same yardstick” as able-bodied counterparts). • *Theme 2: Building Up- Attitudes and Beliefs* (e.g., need to improve quality of life of persons with disabilities; attitude that some occupations or jobs are beyond the abilities of persons with disabilities, with no reference to accommodations or adjustments; “The job itself limits them. Certain disabilities cannot be accommodated. You can’t have a blind man in “tronk” [Afrikaans for working in jail]”. • *Theme 3- Disjuncture; Disconnection and Deviation*- Shared Norms and Reciprocity (e.g., regulations are not always obvious and those who implement them may not have a clear understanding of why regulations are as they are; disconnect between policy and implementation; different understandings of organisational norms and a disjuncture between “them and us” with respect to the levels of hierarchy in an organisation). • *Theme 4- Silence- Shared Goals and Missions* (e.g., no evidence of a shared mission or goal between employer and people with disabilities, or of consulting on their needs or requirements; knowledge sharing was absent).	Small scale case study limits generalisability; lack of insight into the experiences of people with specific types of disability.
Lindsay et al., 2019 (39) Canada	Qualitative interviews (*N* = 35).	18 employers and 17 youth employees with disability, including five with VI and one with VI and hearing impairment.	Themes identified were: • *Disability discomfort* (e.g., employers described how this often stemmed from a lack of experience of working with people with disabilities). • *Reach beyond comfort zone* (e.g., disability awareness training, business cases for employing people with disability, and shared lived experiences, which helped to break down stereotypes). • *Challenging stigma and stereotypes* (e.g., broadening perspectives, challenging stigma and stereotypes, and minimizing bias and focusing on abilities). • *Disability confidence* (e.g., having a supportive and inclusive work culture, and leading and modelling social change. • *Broadening perspectives and challenging stigma and stereotypes*: ○ An employer described their incorrect assumptions regarding the abilities of an employee with VI, “How in the world can she handle all this accounting work and numbers? I quickly learned this young lady had spent her entire life overcoming barriers of being visually impaired…Our assumptions that her visual impairment would limit her in anyway was totally overcome. We've had so many of those stories” (employer #18). ○ “Many customers are happily surprised being served by a blind person. It's an added benefit and value, a different experience to both the server and the guest” (employer #11).	Difficult to extract results relating to specific disabilities; relatively small sample of employers who were self-selected and already collaborating with community disability agencies who support youth with disability and employers to facilitate employment opportunities.

IAT-BVI, Blindness Competence-Specific Implicit Association Test; EABES, Employer Attitudes Toward Blind Employees Scale.

## Results

### Overview of sources

Nine of the articles identified related to research undertaken in the USA, by McDonnell et al. (20, 25–32), reporting on data gathered from four distinct studies. Three of the articles related to research carried out in Norway (35–37), and two from the same research group in Israel (33, 34). One article each reported research undertaken in Greece (24), South Africa (38) and Canada (39). Twelve of the articles discussed quantitative survey research, two of which employed a vignette design (36, 37), and one of which also collected some open-ended response data (37). Two studies employed qualitative interview techniques to gather data from employers, and another used qualitative interviews as a follow-up to a field experiment. Two studies reported on an intervention study, which explored the impact of a meeting with a vocational rehabilitation (VR) professional on employer attitudes (29, 30). Twelve of the articles focused on VI exclusively, and five referred explicitly to attitudes and perceptions relating to VI, but in the context of considering attitudes of employers towards disability more widely. Key findings from the articles are discussed below, providing an overview of attitudes identified amongst employers towards people and employees with VI, concerns reported by employers, and factors identified as having a potential to impact on employer attitudes.

### Explicit and implicit attitudes towards the abilities of employees with visual impairment

Across the articles reviewed, both explicit (those that are deliberate and conscious) and implicit (those which are automatic and unconscious) attitudes of employers were found to be largely negative or, at best, neutral. McDonnall et al. (25, 26, 28) utilised quantitative tools to assess employer attitudes towards employees with VI. The Employer Attitudes toward Blind Employees Scale (EABES) (40, 41) assesses attitudes on two subscales, (a) productivity or ability and (b) challenges relating to employment of people with VI, generating a score ranging from 0 to 66. McDonnall and Crudden (25), McDonnall (28), and McDonnall and Cmar (26) reported average employer attitude scores of 34.03, 35.50, and 34.53, respectively. These scores equate approximately to the neutral point (neither agree nor disagree) on the scale, indicating “neutral” attitudes. However, given the use of self-selecting samples, these attitude scores are likely to be more positive than might be evident in other employer samples. The high proportion of employers (around a third) who had experience of hiring someone with VI in McDonnall and Crudden (25) for example, may have elevated attitude scores. Still, scores appeared neutral, rather than positive.

McDonnall and Antonelli (20) used their Blindness Competence-Specific Implicit Association Test (IAT-BVI) to measure how long employers took to respond to items and to assess the strength with which certain concepts (in this instance, blind and sighted) were associated with certain attributes (i.e., positive or negative). On average, employers more easily associated competence with sighted people, and incompetence with people with VI. Thus, employers had a strong implicit attitude that sighted people were more competent than people with VI. In the same study, the mean score on the EABES was 34.49, around the middle of the score range (neutral), and similar to other means reported for other employer samples (40, 41). Notably, the research also found that the EABES had a low, non-significant correlation with the IAT-BVI, suggesting a discrepancy between implicit and explicit attitudes, a finding typical across the literature surrounding attitudes towards people with disability (42). This discrepancy is important because of the impact of implicit, unconscious attitudes, on behaviours. In the context of employment, these implicit attitudes may influence hiring practices and interpersonal interactions with potential candidates. At an organisational or institutional level, this discrepancy may perpetuate systemic biases and inequalities. If individuals involved in hiring, and the wider organisations, are not aware of implicit biases, it remains impossible to ensure fairer hiring policies and practices regarding. It appears essential for individuals and organisations involved in hiring to recognise and address the gap between explicit and implicit attitudes, in order to ensure equitable practice, policy and behaviours.

Also employing the IAT-BVI, McDonnall et al. (31) found a significant difference between the implicit attitudes of sighted “blindness professionals” (people who worked with individuals with VI for their job) and employers, *F*(1,770) = 210.26, *p* < .0001, *η*^2^ = .21. Blindness professionals provided a mean score of 0.30, demonstrating a slight automatic association for Sighted with Positive (Competence) and Blind with Negative (Incompetence), compared to a mean score of 0.76 amongst employers, indicating a strong automatic association for Sighted with Competence and Blind with Incompetence. Even those who work closely with those living with VI may have negative implicit attitudes towards these individuals, although it seems that experience of working with people with VI may help to reduce these negative beliefs. Research evidences the engrained nature of societal attitudes regarding the abilities of those with disability and, specifically, VI. Improving employment outcomes for people with VI presents a challenge, given the need to improve not only practice and policy, but also increase awareness and address the entrenched belief systems which surround this VI, and the role of people with VI in society and the labour force.

### Intentions to employ people with visual impairment

Whilst not specifically exploring attitudes, searches identified studies which considered intentions to employ people with VI, which may be a useful indicator of employer attitudes.

Papakonstantinou and Papadopoulos (24)'s study employed its own 15-item closed-question measure focused specifically on intentions to hire and offer opportunities to employees with VI, beliefs regarding anticipated challenges, and reactions of colleagues and customers. Results showed that whilst a greater proportion of employers (36.6%) expressed a positive response towards hiring individuals with VI than negative (24.7%), a large majority (74.8%) demonstrated a negative response regarding intentions to hire these individuals on a full-time rather than part-time basis. Furthermore, a majority had negative responses towards developing job roles specifically for employees with VI (63.5%) and almost a third (28.4%) indicated negative responses regarding offering employees with VI the same opportunities for development as other employees. This, once again, appears to reflect negative beliefs relating to the abilities of individuals with VI to perform at work, as well as limits on the lengths to which employers will go in order to include these individuals in the workplace.

In contrast, the majority (58.6%) demonstrated intentions to participate in a funded programme to create appropriate infrastructure for individuals with VI, and nearly half (48%) gave positive responses regarding intentions to attend a seminar about integrating individuals with VI. Furthermore, 69.5% expressed positive intentions to undertake actions to support this integration. Thus, whilst a majority provided negative or neutral answers for most of the questions about hiring intentions and perceptions of people with VI, there was positivity towards engaging with information and initiatives to improve experiences of employees with VI. Notably, reported intentions do not necessarily reflect real-life intentions and actions, and it is not known what the tangible impact of such programmes may be on either attitudes or actions of employers.

In their work to develop the Service from People With Visual Impairment (SPVI), a 6-item psychometric measure of employers’ attitudes towards the abilities of people with VI to perform outward-facing work activities (e.g., interact with customers and suppliers), Eckhaus and Krisi (34) found that 46.8% (*n* = 485) of respondents strongly agreed with the statement “Organizations/employers would prefer employing a person without a disability over a visually impaired or blind person.” This suggests that discriminatory attitudes are viewed as commonplace amongst employers, and at an institutional level.

Research utilising vignettes has also highlighted how people with VI are typically viewed less favourably than potential employees with other types of disability. Fyhn et al. (37) used vignettes to explore employer (*n* = 305) and employee (*n* = 925) assessments of, and willingness to include, ten job seekers with different forms of disabilities (which included a person with auditory impairment, a person with VI, and a wheelchair user), health conditions, or from a minority ethnic background. The vignette describing VI was least likely to be rated positively by respondents, which may reflect particularly negative attitudes towards VI, and/or the low level of experience of working with colleagues with VI in the sample (*n* = 852, 8%). Employers and employees generally agreed in their assessments.

Similar findings are reported by Berre (36), who asked employers to evaluate hypothetical job-seeker descriptions which were manipulated by age, gender, education, work experience, disability, support measure and part-time work. Those with disability were assessed as less employable than those without disability. Furthermore, factors found to impact on assessments for people without disability, namely having more relevant education and work experience or wanting to work part-time, had considerably less, or no impact, on assessments of individuals with disability. Candidates with VI were assessed as least employable, and significantly less employable than any other type of disability. Also, the inclusion of support measures into the vignettes had limited or no effect on hiring assessment scores; marginal effects of VI on employers’ hiring assessment scores were −3.4 for candidates with VI, and just slightly less (−3.2, a non-significant difference) for candidates with VI who had support measures in place (i.e., an accommodation grant), compared to someone with no disability. The research suggests that despite relevant education, experience, and evidenced support measures, a person with VI tends to be viewed as a poorer candidate than most others.

### Benefits of employing people with visual impairment

The majority of sources identified negative attitudes amongst employers, however, qualitative research by Lindsay et al. (39) found that employers of people with VI acknowledged the benefits associated with their employment. Semi-structured interviews with 18 employers and 17 young employees with disability reported on comments from two employers of someone with VI. The first reflected:

How in the world can she handle all this accounting work and numbers? I quickly learned this young lady had spent her entire life overcoming barriers of being visually impaired … Our assumptions that her visual impairment would limit her in anyway was totally overcome. We’ve had so many of those stories (p. 15–16).

A second employer noted the positive impact of interactions between an employee with VI and customers for both parties: “Many customers are happily surprised being served by a blind person. It's an added benefit and value, a different experience to both the server and the guest” (p. 16). Comments highlight the need to challenge stigma and stereotypes for people with disability in work. Broader findings included reflections on benefits of including employees with disability to workplace culture, but also discomfort in relation to disability, often stemming from a lack of experience of their employment. This was overcome by employers in the study, who reported reaching beyond their comfort zone to develop their confidence working with people with disability.

### Concerns reported by employers

#### Capabilities, performance and supporting employees with visual impairment

Concerns over how a person with VI will perform tasks at work were highlighted by two sources. In qualitative interviews run by Ebrahim et al. (38), with two employers of trainees with disability, one respondent reflected on the belief that some occupations or jobs are simply outside the practical capabilities of a person with disability and, in this instance, VI: “The job itself limits them. Certain disabilities cannot be accommodated. You can’t have a blind man in [jail]” (p. 324). Reflecting this, McDonnall and Crudden (25) found that a large majority of employers surveyed (82.3% of 379 hiring managers) did not know how any of the work tasks listed (which included accessing pre-printed materials, using the internet or email, or handling a cashier position) could be performed. Similarly, Papakonstantinou and Papadopoulos (24) reported that most employers surveyed expressed reservations, firstly, for offering voluntary work to potential employees with VI (12.0% gave a negative, and 42.7% a neutral response), and secondly, for offering opportunities for advancement, similar to colleagues without VI (28.4% gave a negative, and 22.6% a neutral response). This, they suggest, likely reflects employers’ fears regarding the work performance of those with VI.

Fyhn et al. (37) found that knowledge of both the individual's ability and accommodations which might help the individual to perform at work, were frequently cited as concerns. This vignette survey study found that the most frequent reason given (from a pre-defined list of options) for a neutral or negative rating of a vignette about a candidate with VI was “accommodation”. Analysis of additional “Other” responses found that “assumptions about accommodations” were made in 14% of responses. These open-ended responses highlighted “Nature of the work” as the greatest perceived barrier relating to those with VI (55%). One supervisor reflected, for example, on the difficulty of “Selling products with visual details”. Findings confirmed that a lack of knowledge about the application of different aids for people with VI may be a key barrier to their employment; this could, in turn, increase concerns regarding the abilities of individuals with VI to perform at work. This highlights the role of educating employers and organisations regarding the equipment, technology and adaptations that might be utilised by someone with VI in order overcome concerns regarding their ability to fit in and perform in the workplace.

#### Customer experience and communication

One article, Papakonstantinou and Papadopoulos (24) reported on concerns regarding customer attitudes towards employees with VI. Their survey of 196 employers found that 13.4% expressed a negative attitude towards the anticipated reaction of customers regarding the employment of someone with VI, and 38.7% a positive response. Such concern may be seen to reflect a lack of knowledge amongst employers regarding if, and how, hiring employees with VI will benefit or harm their business and customer satisfaction. Negative responses may also partly reflect perceived communication difficulties expressed in the study; 20.2% of employers expressed a negative response regarding anticipated communication problems with customers. Yet, 79.8% expressed a positive response regarding communication with customers, suggesting that this concern was not widespread and that other factors may be of greater concern.

#### Cultural “fit” and the experiences of colleagues

Data from two papers suggest that employers may have concerns about the way that employees with VI will fit into, and communicate, at work. In contrast to communication with customers, Papakonstantinou and Papadopoulos (24) found that anticipated communication problems with other employees was a concern for a majority of employers; 81% of employers gave a negative response about anticipated communication problems with colleagues, and greater still, 94% gave a negative response regarding anticipated communication issues with administrative staff at the organisation. Beyond practical concerns that might arise through communication difficulties, findings from Østerud (35) suggest that a lack of cultural “fit” might also be a concern regarding employees with VI. This qualitative exploration of discrimination against jobseekers with disability found that for one employer of someone with VI and impaired mobility, there was a perceived lack of shared work ethic between this individual and their colleagues. This employer suggested that the employee with VI had demonstrated a negative attitude at work, which focused too heavily on difficulties and challenges, and contrasted the positive approach of the rest of their team.

### Factors impacting on the attitudes of employers

#### Age and gender

Three studies reported on the impact of age on employer attitudes towards people with VI. Focusing on implicit attitudes in 343 employers, of whom 14.6% (*n* = 50) had hired someone with VI in the past, McDonnall and Antonelli (20) found that gender and age made little or no difference to employers’ attitudes towards the competencies of employees with VI. This time focusing on explicit attitudes, McDonnall and Crudden (25) reported that in a sample of 379 employers, of whom 32.7% had hired someone with VI and 56% had a personal relationship with someone with VI (friend, family member or neighbour), age was not a significant predictor of employer attitudes. Similarly, Papakonstantinou and Papadopoulos (24) found that age did not appear to impact on employer's attitudes toward hiring people with VI. However, age was a significant predictor of their intentions to participate in a funded program to provide appropriate infrastructures for employees with VI, and to attend an informative seminar regarding labour integration. The older the employer, the less likely they were to participate in these activities. Thus, age may impact on engagement with activities which could improve employment outcomes for people with VI, even if attitudes themselves are not impacted.

The same three studies explored the potential impact of gender on employer attitudes. Whilst McDonnall and Antonelli (20) found no impact of gender on implicit attitudes, the other two studies identified this as an influencing factor. McDonnall and Crudden (25) reported that females in hiring positions had significantly more positive attitudes than males, whilst Papakonstantinou and Papadopoulos (24) found that females were twice as likely to report intending to attend an informative seminar regarding labour integration, and to anticipate positive rather than negative/neutral reactions of customers to the employment of individuals with VI, compared to males. As with age, gender was not found to impact on attitudes themselves in this study.

#### Educational level of employers

McDonnall and Antonelli (20) found that education level of employers made little or no difference to employers’ implicit attitudes towards the competencies of employees with VI. Similarly, McDonnall and Crudden (25) found that education (dichotomised as having or not having a college degree) was not a predictor of employer attitudes. Papakonstantinou and Papadopoulos (24) found that educational level did not impact on intentions to employ people with VI, although, a lower level of education (up to secondary education) was associated with a greater likelihood of a negative response regarding intentions to offer to VI employees the same opportunities as other employees (probability of a positive response was three times lower). Similarly, when education level was lower, the probability of an individual giving a positive instead of a neutral response regarding intentions to participate in a funded program to create appropriate infrastructures for people with VI was two times lower.

#### Size of organisation

McDonnall (28) found that company size (dichotomised by “large” employer, of 500 or more employees, or not) was not a predictor of employer hiring decisions relating to people with VI. McDonnall and Antonelli (27) however, found that company size was associated with the employment of people with VI; employers from large companies (1,000 + employees), and companies with policies, were more likely to hire people with VI. The authors also reported a significant interaction between company size and company policy, indicating that the effect of company policy is dependent on company size. For large companies, having a company policy was not associated with hiring, but for small/medium companies (1–999 employees), having a company policy significantly increased the odds of hiring someone with VI.

Papakonstantinou and Papadopoulos (24) did not specifically explore the impact of the size of company on attitudes but instead found that type of business entity did not influence employers’ attitudes. The authors suggest that this indicates that size of organisation was unlikely a factor, with partnerships and single traders tending to be smaller in size, and other companies and joint ventures tending to be larger. However, in this study, sample make-up meant that responses had to be grouped (negative-neutral), making it difficult to explore the impact on positive vs. negative intentions and attitudes in the study.

#### Relationships and social contact with people with visual impairment

McDonnall and Crudden (25) did not identify personal relationships with people who have VI as a predictor of employer attitudes, but McDonnall and Cmar (26) reported that the odds for employing someone with VI were 3.37 times higher amongst those employers with a personal relationship with someone with VI, compared to those with no personal relationship. Reflecting the latter, Papakonstantinou and Papadopoulos (24) found that lower levels of social contact with people with VI amongst employers (i.e., no contact or one to two times per year or per semester, compared to contact almost every day or one to two times per week or per month) resulted in a greater likelihood of a neutral response than a positive response regarding intentions to hire people with VI.

#### Experience of employing people with visual impairment

As with some of the evidence relating to personal relationships, contact and experience with employees with VI at work has been found to impact on employer attitudes. McDonnall and Crudden (25) found that, of all factors explored (employer knowledge, belief in knowledge, communication with a VR professional, relationship with a VR agency, personal and working relationships with, and previous hiring of, someone with VI), employment of someone with VI in the past was the greatest significant predictor of employer attitudes relating to employees with VI. McDonnall and Cmar (26) report similarly on the direct relationship between both having hired someone with disability in the past and awareness of people with disabilities in the workplace, and more positive employer attitudes. Fyhn et al. (37) also found that previous experience of working with someone similar to a vignette character was associated with more positive assessments of these characters, including the character with VI (*p* = <0.001).

Focusing on implicit attitudes, McDonnall and Antonelli (20) found that employers who had hired someone with VI (*n* = 47) all rated the performance of these employees as average or above average, with those employers who rated performance as above average having significantly more positive implicit attitudes than those who rated performance as average. However, even those employers who rated performance as above average had, overall, a moderate automatic association for Sighted with Positive and Blind with Negative. This suggests that positive experiences with employees who have VI, alone, does not guarantee positive employer attitudes.

Exploring implicit attitudes amongst employers and sighted “blindness professionals”, McDonnall et al. (31) found that type of profession (e.g., teacher of students with VI; vision rehabilitation therapist; orientation and mobility instructor etc.) did not significantly impact on attitude scores, but a comparison of those who had worked in the field for less than, or more than, 17 years found a significant difference between the groups, *F*(1,320) = 3.99, *p* < .05, *η*^2^ = .01. This suggests that additional experiences with people with VI throughout their careers had helped to shape attitudes amongst these professionals, however, no such consideration of time working with employees with VI was made amongst employers in the study.

#### Vocational support and workplace policy

McDonnall (28) found that communication with VR was a significant predictor of hiring decisions relating to individuals with VI. Employers who had communicated with VR had 24.1 times higher odds of having hired a person with VI, compared to those who had not. Reporting on data from the same fieldwork, McDonnall and Crudden (25) found that, in contrast, communication with a VR agency did not predict employer attitudes, but an ongoing relationship with VR was a significant predictor. The authors also identified a correlation between communication with VR and having hired someone with VI, finding that hiring someone with VI in the past was a mediator between communication with VR and employer attitudes. These results suggest that communication with VR influences the hiring of someone with VI which, in turn, influences employer attitudes towards these employees.

McDonnall and Cmar (26) also reported greater odds of hiring someone with VI for those who had communicated with VR in their sample of 387 employers, although these odds were lower than those reported by McDonnall (28). Odds of hiring were 5.61 times higher for employers who communicated with VR compared to respondents who did not (26). Odds of hiring were also 3.80 times higher for those that reported having a company policy about hiring people with disabilities than those that did not (26). However, both communication with VR and company policy were identified only as indirectly impacting on employer attitudes. For example, company policy had an indirect relationship with attitudes through its relationship to hiring and awareness of people with disabilities. This suggests that policy may prompt employers to hire people with VI but does not directly influence attitudes about these employees. Findings from McDonnall and Crudden (25) and McDonnall and Cmar (26) suggest only an indirect impact of communication with VR and company policy on employer attitudes towards people with VI.

#### Knowledge and belief in knowledge regarding visual impairment and capabilities

Across the literature identified, employer knowledge and understanding relating to VI and abilities of people with VI was highlighted as influencing attitudes and perceptions of employability. McDonnall and Antonelli (20) found that those employers with greater knowledge about how people with VI can perform at work were less likely to automatically associate competence with sighted people and incompetence with blind people. Similarly, significant associations were found by McDonnall and Crudden (25) between employer attitudes and both their knowledge about how people with VI perform work tasks, and their belief in their knowledge (i.e., confidence that there is a way for an employee with VI to perform a given task). McDonnall and Cmar (26) also identified knowledge, along with inaccurate beliefs in knowledge, as a predictor of employer attitudes. In this study, whilst many employers indicated that they knew how a person could perform specific tasks (as in McDonnall and Crudden (25), they did not provide an accurate answer as to how they would do so. Those who had hired someone with VI previously were more likely to have a belief in their knowledge but were not necessarily more likely to have accurate knowledge. This may indicate partial, superficial, or forgotten knowledge amongst employers, but also their belief that a range of tasks can be performed by employees with VI, even if their understanding of how might be limited.

Reflecting the above, McDonnall and Antonelli (30) found that a brief intervention, which consisted of an hour-long one-on-one meeting between a VR professional and employer, improved employer attitudes towards, knowledge of, and intentions to hire, people with VI. The study also explored whether delivery of the intervention by a VR professional with or without a VI would impact on outcomes, and whether format of delivery (a dual customer approach or an educational approach) would impact on employer knowledge. Neither variable impacted on improvements in attitudes or intent to hire, and all conditions were effective in increasing knowledge about how an employee with VI could perform work tasks. Notably, for those who received the dual-customer intervention, improvements in attitude scores were significant from pre- to post-test, but not pre-test to follow-up. In contrast, the educational intervention resulted in significant increases from both pre- to post-test and pre-test to follow-up. The educational approach appeared to increase retention of knowledge over time, which may reflect a greater initial gain in knowledge. This same intervention was also found to impact on implicit attitudes, measured by the IAT-BVI. Scores decreased over time across the sample following the intervention [*F*(2, 106) = 2.71, *p* = .07, partial *η*^2^ = .05] (an alpha level of.10 was used to determine significant change in the study), from *M* *=* 0.80 at pre-test to *M* = 0.69 at post-test, and *M* = 0.68 at the 4-month follow-up. As with explicit attitudes in McDonnall and Antonelli (30), neither the type of approach nor the vision status of the VR professional were associated with IAT-BVI scores, although employers who met with a VR professional with VI had a significant decrease in scores over time [*F*(2, 54) = 3.14, *p* = .05, partial *η*^2^ = .10]. It should be noted, however, that even at follow-up, the sample's average score of 0.68 still fell within the strong implicit bias range.

Seeking to integrate employers into the process of addressing negative employer attitudes towards potential employees with VI, Eckhaus (33) utilised survey methods to gather primarily qualitative data from employers, asking them what they felt would encourage an employer or business to consider a candidate with VI. A quantitative measure of attitudes (a Likert-style question) asked employers their opinion on whether an employer would prefer employing a person without a disability over someone with VI. Qualitative analysis identified interactions between four factors and attitudes, all of which highlighted the importance of knowledge and information regarding the abilities and performance of potential employees with VI. Firstly, the impact of evidence of perceived capabilities of the candidate on attitudes. This included seeing other organisations at which people with VI had been employed, and verification of candidate's abilities from a previous employer. Secondly, financial incentives. Third, the interaction between financial incentives and the candidate (i.e., the impact of impairment on the performance of the candidate), and finally, the interaction between financial incentives and information/attitude (i.e., the need for information regarding solutions to potential challenges which might impact on productivity, and the inessentiality of financial incentives where information regarding the candidate and their ability to meet performance requirements is available). Central to three of these themes is the importance of evidencing job capabilities, something that the authors conclude may be of even greater importance to the hiring success of individuals with VI than for candidates from the general population.

#### Implications of employer attitudes on intentions to hire

In addition to exploring employer attitudes and identifying factors impacting on these, several sources reported on the implications of attitudes for hiring outcomes. McDonnall (28) reported that a 10-point higher score on the EABES (40, 41) resulted in 2.61 times higher odds of having hired someone with VI. This in itself does not evidence causality, although McDonnall and Antonelli (27) found that employer attitudes, along with having a personal relationship with someone with VI, were significant predictors of intent to hire people with VI. Data from the same sample (32) found that, of four variables explored by their Theory of Planned Behaviour model (attitudes, subjective norms, behavioural control, and intent to hire), attitudes about productivity of employees with VI had the strongest relationship with intent to hire, followed by subjective norms (perceptions of whether an employer's company and colleagues would support the hiring of someone with VI) and perceived behavioural control (perceptions of authority and ability to hire an individual with VI). Attitudes about perceived challenges associated with employing a person with VI was not significantly associated with intent to hire, although the authors note that that this may reflect the crossover of items in this factor with subjective norms and behavioural control.

## Discussion

The aim of this review was to examine what the existing literature tells us about the attitudes of employers towards employees or potential employees with VI. The systematic searching process identified literature reporting on the explicit and implicit attitudes of employers towards employees, or potential employees, with VI, their intentions to hire these individuals, the concerns expressed by employers regarding their employment, and the factors which might impact on their attitudes. A relatively small number of sources were identified, indicating that this topic is one to which only limited attention has been given in research. Furthermore, most of work in this area has been undertaken in the USA. Whilst comparable to other countries, the USA has its own health and social care system, and a work culture that likely differs to elsewhere in the world. Research which considers the attitudes of employers in other countries will be essential in addressing the unique employment challenges that may be experienced by individuals with VI around the world.

The research identified in the current review highlights several important findings, which may benefit from further investigation. Firstly, there is a prevalence of negative or, at best, neutral attitudes amongst employers towards people with VI. The implications of this are apparent, with current figures for the employment of people with VI being lower than those of other types of disability, and the wider general public. Furthermore, this offers confirmation for the often cited attitudinal barriers experienced by people with VI regarding employment, synthesising evidence which should be considered in the development of hiring and employment practice and policy. Thus far, studies in this area have tended to employ self-selecting samples, who have been aware of the purposes of the research in which they are participating, and often have experience of working with people with VI (25). Even then, attitudes are typically not positive. Exploration of attitudes amongst employers within the wider general population and, importantly, samples that are not self-selecting, have low levels of experience working with employees with VI, and are unaware of the purpose of the research being undertaken, could provide a more representative picture of attitudes across employers.

As in the wider literature surrounding attitudes towards people with disability (42), there appears to be a discrepancy between implicit and explicit attitudes of employers relating to people with VI. Research has shown that whilst explicit attitudes may indicate little prejudice towards people with disability, implicit attitudes typically reveal greater levels of prejudice (43, 44). VanPuymbrouck et al. (44) found that a majority of healthcare providers reported being unbiased against people with disabilities, but implicitly, the overwhelming majority were found to be biased. The authors note the implications of this on equitable access to healthcare and on health outcomes for people with disability. In the context of employment, implicit attitudes may impact similarly on employer decisions regarding people with VI, and ultimately, their employment outcomes. This is concerning given evidence that even where explicit attitudes make progress towards neutrality or positivity, it may be harder to alter implicit attitudes, particularly in relation to disability (45). Research should seek to understand both explicit and implicit attitudes of employers relating to people with VI, including how interventions might address both, and should acknowledge and mitigate, as much as possible, the potential impact of social desirability in research on this topic. Such research would be invaluable in informing educational materials to help employers and organisations evaluate implicit bias within their hiring teams and the wider workforce, to ensure fair access to job opportunities, and inclusive workplace environments.

Previous literature has highlighted a tendency for employers to express even greater concerns about hiring individuals with VI than those with other types of disabilities (46, 47). In the UK, this is reflected in figures showing that of all those people who have disability, people with VI are amongst the least likely to be employed (4). Vignette research suggests that the same disadvantage is also reflected in employers’ attitudes (36, 37). As Berre (36) proposes, the degree of perceived organisational, job, and social misfit from employers appears to vary with type of impairment, and individuals with VI are typically viewed less favourably than those with other types of disability. Even where support measures are in place, employers still appear to view candidates with VI negatively and, in a trade-off between considering a candidates’ qualifications and experience or their disability, disability appears to be the dominating factor influencing employer attitudes and decisions (36). Once again, this may reflect a lack of understanding amongst employers regarding how employees with VI might perform specific tasks at work, indicative of associations between VI and loss of independence (48), and wider negative public perceptions of VI (49). Central to the question of how employment opportuinities can be improved for people with VI, then, is the broader, arguably more challenging question of how negative society-wide attitudes regarding VI, and disability more generally, can be overcome. As research in the current review suggests, perhaps it is that first experience of hiring someone with VI, growth in knowledge, and subsequent increase in confidence amongst employers, that holds the key to eventual hiring and employment equality. Indeed, prior contact with people with VI has been found to positively impact on employer attitudes and intentions to hire individuals with VI (24), particularly where employers have previous experience of employing people with VI (25, 26, 39). As Lindsay et al. (39) suggests, the employment of people with VI, and other disability, may play an important role in breaking down stereotypes relating to disability and, specifically, VI, amongst the wider public. Funding and educational iniatives may play an important role in ensuring that employers take that first step towards the inclusion of employees with VI.

The current review identified several concerns expressed by employers. These related to the ability of employees with VI to perform at work, reactions of customers, the ability of employees with VI to communicate with customers and other employees, and in one study, the cultural “fit” of employees with VI. Papakonstantinou and Papadopoulos (24) propose that one of the main causes of negative or neutral attitudes amongst employers is likely to be a lack of information relating to whether hiring employees with an impairment will benefit or harm them financially. This was reflected in their study by positive attitudes towards employment of people with VI where subsidies or tax exemptions are available, and towards participating in state funded programs to develop infrastructure to support employees with VI. Across the literature, employer knowledge regarding VI and its impact at work was highlighted as influencing explicit and implicit attitudes (20). This evidences the role that education and support may play in overcoming negative attitudes and assumptions made by employers. Indeed, research identified in the current review found that a brief educational intervention improved explicit and implicit employer attitudes towards people with VI (29, 30) but also highlighted the persistence of negative bias towards people with VI amongst employers (30). Earlier research in Greece (50) found similar success in improving employer attitudes through the use of an informative program (a structured booklet outlining characteristics of people with VI, their capabilities, and adaptations in the workplace). Future research exploring the efficacy of different types of intervention to improve attitudes is needed, particularly given the limited geographic spread of existing research. For example, the current review found evidence that support from VR professionals and company policy focusing on disability are associated with hiring decisions relating to people with VI (25, 26, 28). Attitudes might also be impacted, with an ongoing relationship with VR creating opportunities to influence employer attitudes and reduce concerns through consistent support (25). However, ongoing VR support had only an indirect impact on attitudes in McDonnall and Cmar (26). Further research in this area is needed to understand how, and to what extent, employer attitudes and/or hiring behaviours are influenced by educational and vocational support over time, and what the tangible outcomes of this might be for candidates (e.g., increased hiring of people with VI) and existing employees with VI (e.g., greater professional development support, or implementation of VI-awareness training for colleagues).

Several individual factors were also identified as influencing employer attitudes towards people with VI. Results relating to age and gender appear inconclusive regarding direct impacts on attitudes, but there is some evidence that being female and being younger may be associated with more positive attitudes, and with greater intent to participate in activities which might improve employment outcomes for people with VI (24, 25). In contrast, the educational level of employers was identified as unlikely to influence employer attitudes (20, 24, 25). There is mixed evidence regarding the impact of size of organisation on the likelihood of hiring people with VI (27, 28). Further research is needed to explore the impact of this factor, including its potential association with both explicit and implicit employer attitudes, and what other factors (e.g., workplace culture, available accommodations, disability training) might interact to influence attitudes of both employers and colleagues of employees with VI.

It should be noted that contrary to the overall negative attitudes reported, some of the research identified in the current review highlighted positive attitudes of employers. Papakonstantinou and Papadopoulos (24), for example, identified positive attitudes towards intentions to participate in activities which might increase support for, and integration, of potential employees with VI. However, it is impossible to say whether reported intentions are acted upon by employees, and the same research found that intentions to offer targeted job opportunities, or the same opportunities for progression as other employees, were largely viewed negatively. Lindsay et al. (39) reported on qualitative data relating to positive views on the employment of people with disability, including those with VI, although this work was small-scale, and provides insight into only the attitudes of those with first-hand experience of employing people with VI. Future research employing qualitative methods and focusing on the experiences and attitudes of employers of individuals with VI, specifically, would help to increase knowledge in this area. This could help to establish best practice and inform the development of educational materials and interventions to increase knowledge amongst those making hiring decisions and individuals and organisations who do, and do not, employ people with VI.

Findings from this review confirm employee perceptions of negative employer attitudes towards the employment of people with VI (9, 11, 12). This is the first review to focus on this topic from the perspective of the employer, rather than the perspective of individuals with VI themselves. Thus far, there has been little attempt to bring together the narratives of these two groups in research and there is a current lack of consideration of their shared experience in discussions of best practice. Such research may prove valuable in increasing understanding of how employer attitudes impact on employees with VI and their employment experiences, how the behaviours of, and sharing of knowledge from, employees with VI might influence employer attitudes and behaviours, and how the needs of both groups might be met through knowledge sharing, targeted interventions, and training.

## Limitations

The current literature review only included articles published in English, and as highlighted above, identified articles from only a small number of geographic areas. Findings may, therefore, not be globally representative. Whilst articles were sought which explored attitudes of employers, the way that “attitude” was conceptualised and measured varied, including both explicit and implicit measures and qualitative explorations. This may partly explain the mixed findings evidence in relation to the factors impacting on attitudes.

Perhaps of greatest importance is the publication date parameters put on the search. This was considered useful to ensure that the review reflected the most up-to-date research which reported attitudes which were as close as possible to those held by employers today. Still, it is possible that further literature has been published since the completion of this review, and similarly, research carried out prior to 2018 may provide additional insight into the topic at hand, particularly in terms of tracking potential changes in attitudes over time, and how changes in law and policy may have impacted on the experiences of employees and potential employees with VI.

Finally, the review identified only a small number of articles of relevance. This suggests the paucity of research in this field, although it is possible that additional items may have been identified through the inclusion of a greater number of databases. However, the single additional item retrieved in the second and third searches suggest that the results of the review are comprehensive. Thus, whilst this review offers valuable insight into existing literature, it also demonstrates the need for further research, particularly that which explores and compares the attitudes and experiences of both employers and employees.

## Conclusion

This article has outlined current knowledge relating to the attitudes of employers regarding individuals with VI as employees. Findings highlight that whilst these are often negative, processes and interventions to improve knowledge and understanding of VI amongst employers, and how people with VI can successfully contribute at work, may improve attitudes, hiring behaviours, and, ultimately, employment outcomes for people with VI. Whilst this scoping review is not able to make policy recommendations, due to the limitations highlighted above, and the paucity of research undertaken worldwide, it is hoped that this review offers a valuable starting point for future research, and discussion amongst researchers, policy makers, rehabilitation professionals, employers, and those living with VI.
